# West syndrome: a comprehensive review

**DOI:** 10.1007/s10072-020-04600-5

**Published:** 2020-08-22

**Authors:** Piero Pavone, Agata Polizzi, Simona Domenica Marino, Giovanni Corsello, Raffaele Falsaperla, Silvia Marino, Martino Ruggieri

**Affiliations:** 1 Unit of Clinical Pediatrics, AOU “Policlinico”, PO “G. Rodolico”, University of Catania, Catania, Italy; 2 Chair of Pediatrics, Department of Educational Sciences, University of Catania, Catania, Italy; 3 Unit of Pediatrics, Neonatology and Neonatal Intensive Care, and Pediatric Emergency, AOU “Policlinico”, PO “San Marco”, University of Catania, Catania, Italy; 4 Unit of Pediatrics and Neonatal Intensive Therapy, Department of Promotion of Maternal and Infantile and Internal Medicine Health, and Specialist Excellence “G. D’Alessandro”, University of Palermo, Palermo, Italy; 5 Unit of Rare Diseases of the Nervous System in Childhood, Department of Clinical and Experimental Medicine, Section of Pediatrics and Child Neuropsychiatry, University of Catania, AOU “Policlinico”, PO “G. Rodolico”, Via S. Sofia, 87, 95128 Catania, Italy

**Keywords:** West syndrome, Infantile spasms, Epileptic spasms, Infantile spasms syndrome, Etiology, Genetics

## Abstract

Since its first clinical description (on his son) by William James West (1793–1848) in 1841, and the definition of the classical triad of (1) infantile spasms; (2) hypsarrhythmia, and (3) developmental arrest or regression as “West syndrome”, new and relevant advances have been recorded in this uncommon disorder. New approaches include terminology of clinical spasms (e.g., infantile (IS) vs. epileptic spasms (ES)), variety of clinical and electroencephalographic (EEG) features (e.g., typical ictal phenomena without EEG abnormalities), burden of developmental delay, spectrum of associated genetic abnormalities, pathogenesis, treatment options, and related outcome and prognosis. Aside the classical manifestations, IS or ES may present with atypical electroclinical phenotypes (e.g., subtle spasms; modified hypsarrhythmia) and may have their onset outside infancy. An increasing number of genes, proteins, and signaling pathways play crucial roles in the pathogenesis. This condition is currently regarded as a spectrum of disorders: the so-called infantile spasm syndrome (ISs), in association with other causal factors, including structural, infectious, metabolic, syndromic, and immunologic events, all acting on a genetic predisposing background. Hormonal therapy and ketogenic diet are widely used also in combination with (classical and recent) pharmacological drugs. Biologically targeted and gene therapies are increasingly studied. The present narrative review searched in seven electronic databases (primary MeSH terms/keywords included West syndrome, infantile spasms and infantile spasms syndrome and were coupled to 25 secondary clinical, EEG, therapeutic, outcomes, and associated conditions terms) including MEDLINE, Embase, Cochrane Central, Web of Sciences, Pubmed, Scopus, and OMIM to highlight the past knowledge and more recent advances.

## Introduction

An increasing and changeable number of terms, on the disorder first reported by William James West (1793–1848) on his own son James Edwin (1840–1860) in 1841 [1], and subsequently labeled as “West syndrome” (WS), have been progressively proposed, first adopting the term infantile spasms (IS) in accordance with the most relevant clinical event, and later the term epileptic spasms (ES), since the disorder may have its onset outside infancy [2, 3].

The classical terms WS and IS are still the most quoted in the literature and, concordantly, the term ES has been incorporated within the umbrella definition of “infantile spasms syndrome” (ISs), as the different seizure types, the EEG and developmental features tend to occur all together and at the same time [4, 5]. The ISs belongs to the group of “early epileptic encephalopathies” (EEE), characterized by severe, drug-resistant epileptic disorders, with onset in early life, associated to persistent EEG abnormalities and cognitive defects. By definition, in this group of disorders, the seizures per se may contribute, above and beyond the causal effects, to the progression of cerebral dysfunction [5–8]. The ictal phenotype is the result of a cellular/molecular cascade of events, which in turn is responsible for the neurodevelopmental abnormalities. The ictal phenomenon, without associated EEG abnormalities, has been reported [9]. In several reports West syndrome, infantile spasms, epileptic spasms, and infantile spasms syndrome (WS, IS, ES and ISs) are still used interchangeably. In this report, we prefer to use the term “IS” to indicate the ictal phenomenon and the term “ISs” to describe the (spectrum of) disorder(s) associated to IS.

Over the last years, new advances in all the relevant and less common aspects of this uncommon disorder have been achieved including its nomenclature, etiology, associated genetic factors, variety of clinical features and complex phenotypes, and modalities of treatment and prognosis [7, 8].

The ISs is estimated to occur in about 0.249 cases/1000 live births [10] with an overall prevalence of 1/10,000 children at the age of 10 years [11, 12]. The number of affected children by this condition is consistent over time and no increase has been recorded in the most recent population-based and case-series studies [10, 11]. Both genders are affected by a relatively small prevalence of males.

In the present review article, we aimed to highlight the past knowledge and more recent advances in this condition by narrative literature review according to the search strategy and methodologies indicated here below.

## Search strategy and methodology

In this narrative review, six online bibliographic databases were searched from inception to May 30, 2020: MEDLINE (from 1946), Embase (from 1946), PubMed (from 1966), Cochrane CENTRAL (from 1996), Web of Sciences (1997), and Scopus (from 2004).

We used an empirical and topical approach to derive an objective primary search strategy for identifying clinical, laboratory, and therapeutic studies of digital interventions that included West syndrome, infantile spasms, and infantile spasms syndrome.

In the first step, we identified a test set of critical papers meeting our broad inclusion criteria by introducing in the search bar the major MeSH term “West syndrome” (18,584 results) OR “infantile spasms” (4569 results) OR “infantile spasm syndrome” (2273 results); the search strategy was developed in MEDLINE using article identification numbers, and the search strategy was iteratively improved to maximize the sensitivity and specificity for identifying relevant articles. This search strategy achieved 100% sensitivity against these initial test sets. Following this step, key search terms derived from the medical subject heading terms used in the test papers were established, which included 12 primary sets of terms pertaining to “West syndrome” OR “infantile spasms syndrome” studies (both terms indicated in all paragraphs here below in parentheses and separated by semicolons) and digital interventions: these relevant terms included (AND) “clinical spasms” (1866; 1061 results), “epileptic spasms” (4601; 2263 results), “electroencephalography” (1807; 868 results), “electroencephalographic” (323; 169 results), “EEG” (1205; 1021 results), “Video-EEG” (59; 49 results), “mental retardation” (1579; 656 results), “developmental delay” (485; 285 results), “developmental regression” (101; 44 results), “intellectual disability” (1541; 538 results), “cognitive” (788; 205 results), and “disability” (1431; 516 results).

We conducted a second search and introduced in the search bar the MeSH term “West syndrome” OR “infantile spasm syndrome” AND “inborn errors of metabolism” (320; 83 results), “brain malformation(s)” (1047; 461 results), “chromosome/chromosomal” (758; 214 results), “neurocutaneous syndromes/disorders” (325–330; 96–99 results) OR “phacomatosis/phacomatoses” (327; 96 results), “immune/immunology”, “immunological” (42–46 results), “infective/infectious” (796; 21 results).

A third search included in the search bar the MeSH term “West syndrome” OR “infantile spasm syndrome” AND “gene” OR “genetics” (767–1800; 432–2263 results), “protein(s)” (4654; 738 results), and “pathway” (4653; 729 results).

An additional, and fourth search included the MeSH terms “West syndrome” OR “infantile spasm syndrome” AND “therapy/treatment” (8169–9487; 1022–1180 results) OR “outcome” (3414; 551 results) OR “prognosis” (3414; 851 results).

A variety of terms related to each of these terms were entered into each database. References of relevant sources, which were manually examined to identify any additional relevant studies, were included in the reference list. After the removal of duplicate records, two reviewers (PP and MR), in their mid/advanced-career research, trained in this method, independently screened the titles and abstracts for relevance, and then extracted and selected relevant full-text records and pivotal papers known to either reviewer. Discrepancies were resolved through discussion at each stage, and a consensus was achieved with acceptable inter-rater reliability.

Notably, when we coupled in our search the MeSH term “West syndrome” vs. “infantile spasm syndrome”, the results were more significant, in all searches carried out, in the former matching, meaning that the classical term West syndrome is still more incardinated and most widely used in the literature.

A separate search was conducted in the OMIM™ (Online Mendelian Inheritance in Man, 2020) database (available at https://www.omim.org) by entering the term “West syndrome” OR “infantile spasm” OR “infantile spasm syndrome”: the main results of this search are listed in Table 1 and discussed throughout the review.

**Table 1:** list of the most frequent genes associated to ISS

Gene	Cytogenetic location
***ARX***	Xp21.3
***CDKL5***	Xp22.13
***PAFAH1B1/LIS1***	17p13.3
***DCX***	Xq23
***TUBA1A***	12q13.12
***STXBP1***	9q34.11
***KCNQ2***	20q13.33
***SPTAN***	9q34.11
***MAGI2***	7q21.11
***GRIN2A***	16p13.2
***FOXG1***	14q12
***NSD1***	5q35.3
***NEDD4***	15q21.3
***CALN1***	7q11.22
***WDR45***	Xp11.23
***SLC1A4***	2p14
***RARS2***	6q15
***UBA5***	3q22.1
***IARS2***	1q41
***PHACTR1***	6p24.1
***ATP2A2***	12q24.11
***CD99L2***	Xq28
***CLCN6***	1p36.22
***CYFIP1***	15q11.2
***CYFIP2***	5q33.3
***GNB1***	1p36.33
***GPT2***	16q11.2
***HUWE1***	Xp11.22
***KMT2D***	12q13.12
***MYO18A***	17q11.2
***NOS3***	7q36.1
***RYR1***	19q13.2
***RYR2***	1q43
***RYR3***	15q13.3-q14
***TAF1***	Xq13.1
***TECTA***	11q23.3
***PURA***	5q31.3

*ARX*, aristaless related homeobox; *ATP2A2*, ATPase sarcoplasmic/endoplasmic reticulum Ca2+ transporting 2; *CALN1*, calneuron 1; *CD99L2,* CD99molecule like 2; *CDKL5*, cyclin dependent kinase like 5; *CLCN6*, chloride voltage-gated channel 6; *CYFIP1*, cytoplasmic FMR1 interacting protein 1; *CYFIP2*, cytoplasmic FMR1 interacting protein; *DCX*, doublecortin; *FOXG1*: forkhead box G1; *GNB1*, G protein subunit beta 1; *GPT2*, glutamic--pyruvic transaminase 2; *GRIN2A*, glutamate ionotropic receptor NMDA type subunit 2; *HUWE1*, HECT,UBA,WWE domain containing 1; *IARS2*, isoleucyl-tRNAsynthetase 2, mitochondrial; *KCNQ2*, potassium voltage-gated channel subfamily Q member 2; *KMT2D*, lysine methyltransferase 2D; *MAGI2*, membrane associated guanylatekynase; *MYO18A*, myosin XVIIIA; *NEDD4*, neural precursor cell espressed, developmentally down regulated 4-2, E3 ubiquitin protein ligase; *NOS3*, nitric oxide synthase 3; *NSD1*, nuclear receptor binding SET domain; *PAFAH1B1*, platelet activating factor acetylhydrolase; *PHACTR1*, phosphatase and actin regulator 1; *PURA*, purine rich element binding protein A; *RARS2*, arginyl-tRNAsynthetase 2, mitochondrial; *RYR1*, ryanodine receptor 1; *RYR2*, ryanodine receptor 2; *RYR3*, ryanodine receptor 3; *SLC1A4*, solute carrier family 1 member 4; *SPTAN*: spectrin alpha, non-erythrocytic

In addition to all of that, we also introduced and commented on the first article by William J. West, 1841 [1].

## Etiologic factors

### ISs—one syndrome (one phenotype) for many different etiologies

The overall spectrum of the so-called ISs, as it occurs for other neurological disorders, including epileptic seizures and cognitive and behavioral developmental disabilities, is caused by different pathogenic events, some of which are still unknown while others are well-recognized structural, infectious, metabolic and immunologic defects and genetic abnormalities [6]. All these factors may act, mostly, as single causal events or in complex associations. Often, an obvious etiologic distinction is not strictly feasible, since different events may concur in causing the ISs. In about 35% of cases, the etiologic event is (still) unknown: the outcome, in these cases, is usually more favorable as compared with the group with recognizable etiologies. Yuskaitis et al. [13], reported in 133 infants with ISs of unknown origin, normal development in 15% vs. clinically well-documented developmental delay in the remaining 85%.

The causes of ISs may have their origin in the prenatal, perinatal, and post-natal period. The hypoxic-ischemic encephalopathy is reported as one of the most common causes of ISs. In the study carried out by the “United Kingdom Infantile Spasms Study” (UKISS) [14], in 127 out of 207 patients affected by ISs with proven etiological diagnosis, hypoxic-ischemic encephalopathy was reported in 10%, followed by chromosomal abnormalities, complex malformation syndromes and perinatal stroke (8%), tuberous sclerosis (7%), and periventricular leukomalacia or hemorrhage (respectively, in 5%). Hypoxic-ischemic encephalopathy, prenatal cerebral infections, and stroke may act causing permanent cerebral damage manifesting subsequently with ISs. It must be noted that many of the etiologic events in the UKISS study [14] are similar from a strict pathogenic viewpoint: e.g., the molecular/cellular events leading to the typical brain defects in tuberous sclerosis (which is regarded as a malformation of cortical development) are similar to the events occurring in some complex malformation syndromes or in chromosomal abnormalities with brain structural defects. That may occur as the cascade of involved genes/proteins belongs to common intracellular signaling pathways. Most importantly, many recent studies highlight that vascular events (of all types: i.e., hypoxic, ischemic, stroke), infections, metabolic and immunologic defects, may act on a genetic predisposing background [15, 16]. More recently, the results of a study carried by the “National Infantile Spasms Consortium” in North America on 161 (64.4%) out of 250 ISs patients in whom the etiologic cause was achieved, revealed that genetic factors were recognized in 14.4%, genetic-structural in 10.0%, structural-congenital in 10.8%, structural-acquired in 22.4%, metabolic in 4.8%, and infectious in 2% [17]. In this respect, according to the widest epidemiologic studies, genetic-molecular involvement has been increasingly recorded in patients with ISs when using modern genetic technologies (i.e., array-CGH, NGS, WES, and WGS), allowing the identification of an increasing number of genes or copy number variants (CNVs) involved in the direct expression of the ISs [15, 16]. The molecular/cellular anomaly may act directly in generating by itself the neuronal/brain structural phenotype underlying the ISs or indirectly by causing a complex syndromic phenotype (i.e., a recognizable malformation syndrome or an inborn error of metabolism) or by predisposing (at the cellular level) to a vascular or infectious event [16]. Genetic causes may be involved in yielding the ISs through several mechanisms: chromosomal or large/single gene abnormalities, or CNV, or a mixture of all of these factors.

### The role of genetics as an etiologic factor

As reported by Scheffer et al. [6], the majority of genes involved in ISs [see Table 1] show a phenotypic heterogeneity, as it occurs with other neurological disorders.

A genetic predisposition to cause ISs was advanced by Dulac et al. [18] and by Hemminki et al. [19], on the basis of the observation that the chance of having ISs was increased in families in which other members were affected by epileptic seizures. A genetic predisposition in cases of ISs was also confirmed by reports of IS in twins. In this latter respect, Pavone et al. [20] first described the occurrence of IS in a set of monozygotic twins who had their onset of spasms within a short interval of time (a few hours) one from the other. Similar findings were recorded by Coppola et al. [21] in three independent sets of monozygotic twins: in each set of twins, the epileptic spasms appeared on the same day, within hours one twin from the other. This phenomenon is difficult to explain: we could only hypothesize that a time-related, pre-programmed molecular/cellular event may act triggering, in a given individual, the onset and the overall occurrence of spasms, somewhat similar to the programmed phenomena of apoptosis and cellular death occurring during life [15, 20]. A different, likely complementary, explanation could be that environmental triggers affect (ISs) genetically predisposed monozygotic twins at the same time [15].

Direct involvement of genes in the etiology of ISs was related to the detection of mutations of the Aristaless-related homeobox (*ARX1*) gene and the Cyclin-dependent Kinase-like 5 gene (*CDKL5*), both located in the human chromosome Xp22 region, in patients affected by complex malformation phenotypes with IS/ES [15, 16]. These two genes are widely expressed in fetal brains and their role in brain developmental has been firmly demonstrated [4, 15, 16, 22–27].

As reviewed by Paciorkowski et al. [16], mutations in other-than-X-linked genes, including *PAFAH1B1/LIS1*, *DCX*, and *TUBA1A* are also frequently associated with ISs [15]. These genes are expressed in GABAergic interneurons and their mutations have been regarded as a direct cause of ISs, secondary to neuronal cell disruption during embryogenesis [8, 16, 24]. Other ISs-causing genes include *STXBP1* [28, 29], *KCNQ2* [30], *GRIN2B*, and *GRIN2A* [31, 32], and *MAGI2* [33]. Further and more recent causative mutations of genes involved in the pathogenesis of ISs include the *SPTAN*, *FOXG1*, and *NSD1* genes [11], thus confirming the wide variety of involved genes causing ISs. A study of Boutry-Kryza et al. [32], on 73 patients with different types of ISs, by means of array-CGH testing and genomic sequencing revealed anomalies in CNVs in multiple genes in up to 15% of patients, including three patients who harbored specific point mutations in *CDKL5* and *STXBP1* genes; they also recorded [32] ISs patients yielding microdeletions in the 2q24.3, 5q14.3, and 9p34 regions, respectively; and microduplications in the 2q24.3 and Xp28.11.93 regions. According to the same study [32], the 16p12.1 deletions recorded in their ISs series, which included intronic deletions of the *NEDD4* gene and intronic deletions of *CALN1* gene, could be regarded as potential risk factors for ISs.

Recent studies have expanded the spectrum of ISs-associated genes, by including mutations in *WDR45* [34, 35], *KCNQ2 R198Q* [36], *SLC1A4* (variant) [37], *RARS2* [38], *UBA5* [39], *IARS2* [40], *hCDKL5* [41], and *PHACTR1* [42] genes. A study on a cohort of 56 Chinese families with ISs screened by means of WES, revealed 17 novel ISs-candidate genes: *ATP2A2*, *CD99L2*, *CLCN6*, *CYFIP1*, *CYFIP2*, *GNB1*, *GPT2*, *HUWE 1*, *KMT2D*, *MYO18A*, *NOS3*, *RYR1*, *RYR2*, *RYR3*, *TAF1,TECTA* and *UBA* [43].

Most recently, ISs children harboring 5q31.2-q31.3 microdeletions (a region embedding the *PURA* (purine-rich element binding protein A) gene), were reported by Shimojima et al. [44]. De novo mutations in the *PURA* gene are responsible for a neurodevelopmental disorder (the so-called PURA syndrome) characterized by severe intellectual disability, epilepsy, feeding difficulties, and neonatal hypotonia associated to respiratory and gastrointestinal problems, eye anomalies, endocrine defects, exaggerated startle responses, hyper somnolence, and hypothermia [45]. These latter findings further demonstrate how wide and complex is the “evolving” spectrum of neurodevelopmental disorders including (typical and atypical) IS in their phenotypes [15, 16].

### Additional causal factors

#### Structural brain disorders

Structural brain abnormalities are well-known causes of IS and ISs: lissencephaly, focal cortical dysplasia, polymicrogyria, hydranencephaly [46], and hemimegalencephaly are some examples of ISs-underlying developmental brain anomalies. Specifically, the *PAFAH1B1/LIS1* and *DCX* genes, which are related to classical lissencephaly, appear to be associated to ISs in about 80% of affected children. A de novo heterozygous mutation of *KIF2A* gene has been reported in a child with lissencephaly, developmental delay, and IS [47]. Recently, a child with IS and periventricular nodular heterotopia was found to harbor an unbalanced chromosomal translocation 3p26.2-10p15.1 and a 6q22.31 duplication [48]. A child with a novel homozygous nonsense mutation in the *B3GALNT2* gene was reported with clinical features compatible with a diagnosis of Walker-Warburg syndrome, ISs, and sensorineural hearing loss [49].

#### Complex malformation syndromes

The Down, Pallister-Killian, and Williams-Beuren syndromes have been often associated to ISs.

In a study of 183 Down’s syndrome patients admitted to our institution in the 1990s, 15 complained of epileptic seizures and among these 4 suffered from ISs [50]. According to Tapp et al. [51], the prevalence of epileptic seizures in patients affected by Down’s syndrome ranges from 1 to 13%: among these 6–32% present with ISs.

Mosaicism for tetrasomy of chromosome 12p is the main cause of Pallister-Killian syndrome: affected patients show skin pigmentation, bitemporal alopecia, rugged-looking face, epileptic seizures, and intellectual disability. Notably, these patients present late-onset epileptic spams [52].

Williams-Beuren Syndrome (WBS) is linked to a chromosomal microdeletion manifesting with characteristic facial features and heart problems associated with intellectual disability and happy and affable behavior. Marshall et al. [33] reported on a WBS patient with ISs harboring a large deletion on chromosome 7q11.23-q21.11 embedding the *MAGI2* gene.

Anecdotal cases of ISs have been reported within the context of Schinzel-Giedon, Smith-Lemli-Opitz, Smith-Magenis, and Sotos syndromes [11].

Epileptic spasms have been also recorded in a male infant with *PPP1CB*-associated Noonan-like syndrome [53].

ISs-associated features are the presenting manifestations of two well-characterized syndromes: the PEHO and Aicardi syndromes. The PEHO syndrome is a rare and progressive encephalopathy presenting with edema, hypsarrhythmia, and optic atrophy with remarkable cerebellar atrophy due to granule neuron loss. Recently a mutation in the *ZNHIT3* gene has been identified as its primary cause [54]. Aicardi syndrome is a neurodevelopmental syndrome affecting the female gender: corpus callosum agenesis, retinal lacunes, severe intellectual disability, and ISs with an EEG showing asymmetric hypsarrhythmia are the main abnormalities; brain tumors, especially affecting the choroid plexus have been also reported [55].

#### Inborn errors of metabolism

Phenylketonuria (PKU) is an inborn metabolic disorder caused by a mutation of the gene encoding for the enzyme phenylalanine hydroxylase (PAH), which converts the amino acid phenylalanine into tyrosine and other components. In the pre-screening era, the PKU incidence was as high as 1 in 5000 newborns and was characterized by skin hypopigmentation, severe developmental delay, and seizures including ISs. In untreated patients, severe brain demyelination and abnormalities in gray matter are responsible for the severe cerebral involvement. A rare but more severe subtype of PKU is a disorder related to tetrahydrobiopterin (BH4) deficit, a coenzyme of PAH. In untreated patients, developmental delay and epileptic seizures of the ISs type are almost constantly reported [56]. Early-onset inborn errors of metabolism may present with ISs as their first manifestation. Alrifai et al. [57] in a group of 80 children presenting with ISs, recorded a diagnosis of neurometabolic disorders in 10 (12.5%): among these, two showed a Leigh-like disorder, the others were affected by ethylmalonicaciduria, non-ketotichyperglycinemia (caused by the *GCSH* gene), hyperinsulinemic hypoglycemia (HHF17), short-chain acyl-coenzyme A, dehydrogenase deficiency (caused by the *ACADS* gene), molybdenum cofactor deficiency (subtypes MOCS12; caused by the *GPHN* gene), primary carnitine deficiency (caused by the *SLC22A5* gene), and neonatal hypoglycemia secondary to hypopituitarism (subtypes CPHD15). ISs may be also a clinical feature in children with glycine encephalopathy (caused by the *GLDC* and *GCST* genes); DEND (developmental delay, epilepsy, neonatal diabetes caused by the *KCNJ 11* gene); methylmalonicaciduria (caused by the *MUT* gene), maple syrup urine disease (caused by the *BCKDHA*, *BCKDHB*, *DBT*, and *DLD* genes) and propionic acidemia (caused by the *PCCA* and *PCCB* genes).

ISs have been also reported in children with neurodegenerative disorders including globoid cell leukodystrophy-Krabbe disease (caused by the *GALC* gene) and Menkes disease (caused by the *ATP7A* gene) [58–60]. Rare disorders associated with ISs also include cerebrotendineous xantomatosis (caused by the *CYP27A1* gene) [61]; glucose transport 1 deficiency (caused by mutations in exon 9 of the *SLC2A1* gene) [62]; disorders of glycosylation [63] (caused by the *ALG1*,*6*,*11* genes: subtypes *CDG* and *CDG 1x*). Pyridoxine-dependent epilepsy (PDE) may present with various types of severe seizures, partial and generalized seizures, atonic and myoclonic seizures, convulsive status epilepticus, and ISs [64, 65]. Mutation in *ALDH7a1* gene encoding the alfa-amino-adipic-semialdehyde (alfa AASA) dehydrogenase (antiquitin) is a well-recognized cause of epileptic seizures including ISs [16, 65]. As reported by van Karnebeek et al. [64], PDE may manifest with atypical features with late-onset and different response to the pyridoxine and metabolic disturbances like hypoglicemic episodes, lactic acidosis, and electrolyte anomalies. Neurotransmitter and neuroimaging abnormalities may precede the onset of ISs [65]. We have followed an infant with ISs clinically and EEG confirmed in whom treatment with large daily supplements of pyridoxine resulted in disappearance of the symptoms. (Fig. 3A, B).

#### Neurocutaneous disorders (*phacomatoses*)

ISs may be one of the (earliest) manifestations in some neurocutaneous disorders or phacomatoses. Within this context, affected children typically show non-casual associations of congenital skin (and eye) anomalies and central (and peripheral) nervous system structural abnormalities (and/or tumors), and neurological manifestations, often associated to systemic involvement (e.g., the heart and vessels, lung, kidney, and bone) [66–68].

#### Tuberous sclerosis complex (TSC)

TSC is the most common example of a neurocutaneous disorder typically associated to ISs [69, 70]. TSC-affected children often (up to > 90% of cases) present with different epileptic seizure types [70]. Mutations in either the *TSC1* or *TSC2* genes, through the hamartin/tuberin complex and mTOR pathway, cause neuronal/interneuronal disruption, which in turn leads to a malformation of brain cortical migration and layering, which is reflected by the development of cortical tubers, white matter anomalies, subependymal nodules (and related subependymal giant cell astrocytoma), and brain cysts [71]. These brain structural abnormalities form the basis of susceptibility to manifest epileptic seizures (and of the overall neurodevelopmental, behavioral, and cognitive defects) [72]. In a study on a population of 81 TSC children with a median age of 10 years, epileptic seizures occurred in 91%, including 32% with a history of ISs [73]. Several studies documented an important decrease (in frequency and severity) vs. cessation of seizures in TSC patients with drug-resistant epilepsy (including individuals with a past history of ISs) treated with focal cortical tuber resection [74, 75]. New treatments with single and/or combined m-TOR inhibitors have been proposed in patients with TSC and (drug-resistant) epilepsy, including ISs, but criticism was raised by the remarkable side effects related to their use and by the relatively poor efficacy over time [76–78].

#### Neurofibromatosis type 1 (NF1)

Patients with NF1 show a genetic predisposition to the development of benign (and, less frequently, malignant) central and/or peripheral nervous system and systemic tumors, which are related to the effect of loss of neurofibromin, the *NF1* gene protein product [66, 68, 79, 80]. In a cohort of 630 NF1 patients, 37 (5.87%) suffered from epileptic seizures: among these, the most common seizures types were partial and primary generalized, with only two children presenting with ISs [81]. In a large multicenter hospital-based study and systematic review of the literature, the NF1 children presenting with IS represented only 1.5% of the NF1 population, most having a more favorable outcome [82]. Seizures, epilepsy, and infantile spasms, within the context of NF1, have been related to disruption in the ras/NF1-related MEK/MAPK/ERK signaling pathway [68, 82].

#### Sturge-Weber syndrome

Capillary vascular malformations of the embryonic facial vasculature, the leptomeninges (including underlying neuronal disarrays) and the choroidal eye layer are the main features of SWS [66, 68, 83]. This complex vascular developmental disruption syndrome is caused by somatic mutations in the *GNAQ* gene, a nuclear structural gene responsible for vessels and neuronal development [84]. Epileptic seizures (as well as cognitive and behavioral abnormalities) are the most frequent clinical manifestations of the disease and are reported to occur in up to 77% of patients with unilateral, and 92% with bilateral brain involvement [68, 85, 86]. The epileptic seizures are mainly of the partial but also tonic-clonic generalized types [86]: ISs have been sporadically recorded [86, 87].

### Pigmentary mosaicism of (the hypomelanosis of) Ito type

Seizures and epilepsy, occasionally including ISs, can be recorded in children (and adults) with skin pigmentary (usually of the hypopigmented type) whorls and streaks following the lines of Blaschko (i.e., a system of lines in the skin reflecting the arrangement and migration patterns of pigmentary cells in the human embryo and later in postnatal life). Affected individuals can present also associated extra-cutaneous abnormalities mostly affecting the eye, musculoskeletal, and nervous systems: this latter complex malformation phenotype is currently known as hypomelanosis of Ito, a condition which reflects somatic mosaicism for some, yet unknown, pigmentary genes) [88, 89]. Epilepsy, and the occurrences of ISs, within the context of hypomelanosis of Ito, is likely due to the minor (mosaic) migration disordering (manifesting as white matter disarray) typically associated to the (more severe) complex neurological phenotype [89, 90].

#### Role of immunity

Recent studies have hypothesized the possible role of immunologic events as triggering factors of ISs. This is related to the observation that some genes, involved in the pathogenesis of ISs, play also crucial roles in a variety of inflammatory cascades and signaling pathways [91]. Lemke et al. [31] reported mutations in the *GRIN2B* gene (which encodes the *NR2B* sub-unity of the N-methyl-aspartate (NMDA) receptor), in two children with ISs: NMDA receptors are involved in a number of neurological disorders [31]. Notably, elevated titers of antibodies against the voltage-gated potassium-channel complex proteins (VGKC) (i.e., 201 pmol/L; normal values = < 100) were reported in an infant aged 4 months with ISs [92].

### Pathogenesis

The overall pathogenesis of ISs may present with many obscure aspects: as previously summarized, a number of factors can cause ISs and thus it may prove difficult to explain each single event causing ISs [15, 16, 93]. There are well-documented examples of ISs originating from whole cortical involvement, as it occurs with lissencephaly, or from focal cortical disarrangements, as recorded in children with polymicrogyria or in individuals with cortical tubers and white matter disarray secondary to TSC, or from subcortical impairment as it occurs in children with hydranencephaly. The most likely scenario could be that of a disruption of the normal brain neuronal/interneuronal network(s) (either at the molecular, receptor, or cellular level) [15, 16] leading in turn to abnormal interactions between cortical and subcortical structures [93]. Another issue that needs clarification is the (apparent) discordance between the general diffuse (EEG) pattern of hypsarrhythmia (coupled to the generalized clinical pattern of spasms) and the focal cortical lesions recorded in many cases of ISs. An hypothesis is that the focal cortical lesions may spread down to the basal ganglia thus providing the basis for making it manifesting both the clinical (generalized) appearance of spasms and the hypsarrhythmia pattern [93–95]. A further issue to clarify is the cognitive/intellectual disability and the autism spectrum disorder (ASD) often associated to (but also preceding the onset of) ISs. Deletions of *SCN2A* and *SCN3A* genes were found in a young boy with autistic spectrum disorder and ISs [96]. These [96], and other findings (reviewed [15, 16]) seem to support the hypothesis of a genetic (predisposing) background leading at the same time to the appearance of IS and the co-occurrence of a mixed cognitive/behavioral defect, including the intellectual disability and the autism spectrum phenotype.

## Clinical features, seizure types, and EEG features

### ISs vs. West syndrome

WS is regarded as a subtype of ISs and is the most frequently reported subtype presenting in about 90% of cases of ISs. WS encompasses the triad of infantile spasms, hypsarrhythmia, and developmental arrest or regression. The classical presentation of WS consists in short episodes of abrupt flexion of the trunk and neck and adduction of the arms, with onset in infancy or early childhood. The tonic spasms are bilaterally symmetric, each lasting few seconds and occurring at wakening and in clusters. EEG recording typically shows a hypsarrhythmic pattern consisting in chaotic mixture of very high amplitude slow waves with discharges of waves and spikes varying in amplitude, morphology, duration, and site. Psychomotor delay or developmental regression is associated features [11, 12].

ES (i.e., the newer term for IS) are regarded as a component of WS, and are clinically defined by abrupt contractions followed by a tonic contraction lasting a few seconds with involvement mainly of the muscles of the neck, trunk, and limbs with abduction or adduction of the arms. The spasms may appear in flexion, extension or in mixed patterns with episodes of cry or scream, which may precede or follow the spasm itself. The spasms appear mostly in rapid sequence and occur prevalently just before sleep or on awaking. In the most severe ictal phenotypes, the spasms may be noticed also during sleep. During the crises, the eyes may be fixed or deviated and there can be cardiac and respiratory involvement. Affected children after the crises may be irritable or may have drowsiness [17, 97, 98]. The spasms may occur in association with episodes of facial grimacing, transient focal movements, and blinking [11, 99]. In general, the onset of spasms occurs between 4 and 9 months with a peak around the 6th month of life: in about 80–90% of cases, the spasms manifest within the first year of life. In the initial phase of the disorder these phenomena may pass unnoticed and overlooked by parents [15]. As reported by Hussain et al. [100] in a study carried out in 100 patients with ISs the treatment was started only after more than a week from the onset of the first clinical manifestations with a median time of delay of treatment of 24.5 days. Wang et al. [101] developed an algorithm using medical claims data at the aim to properly and early identify the ES.

Aside the above reported, classical ictal phenomena, atypical patterns of clinical (and EEG) manifestations of ISs are well known. Atypical patterns may be related to the age of the child at onset of spasms and to the etiologic factors underlying the disorder.

### ISs—atypical presentations vs. association of spasms with other seizure types

IS may present with hypsarrhythmia but without clearly definable clinical signs: these spasms are indicated as “Subtle Spasms”. Slow abnormal limbs movements, facial grimacing, isolated fixed eyes, slow trunk rotation may be the only clinical expressions of ISs and can be associated to other seizure types.

Xue et al. [102] recorded, in 12 ISs children, three different patterns of atonic spasms combined or uncombined with typical IS: spasms-atonic, pure atonic, and atonic-spasms seizures.

In a study on 48 patients presenting with IS in clusters without hypsarrhythmia Caraballo et al. [103] was able to recognize two subgroups: (1) in 30/48 patients he recorded a well-defined electroclinical syndrome with IS manifesting mainly in infancy; (2) in 18/48 there were variable patterns of electroclinical syndromes manifesting as epileptic encephalopathies other-than-ISs, as the main clinical manifestation; among these 48 patients, nine had electroclinical features of Lennox-Gastaut syndrome, four myoclonic and atonic seizures, two were affected by Dravet syndrome of whom one presented with epilepsy of infancy with migrating focal seizures. Other children showed non-convulsive status epilepticus with atypical absences and also clinical features of sub-acute sclerosing panencephalitis.

“Single ES” are defined as no other spasms occurring for 1 min before and after each other. A group of 16 children with this clinical phenotype was clinically evaluated by Caraballo et al. [104]: 9/16 patients showed a hypsarrhythmic EEG pattern, which was not recorded in the remaining 7/16. Overall, in these patients, other types of seizures were also recorded, both before and during their epileptic spasms. An infant with a triad of a clusters of IS, vertical binocular nystagmus, and focal tonic seizures manifested during a single ictal event was reported by Tarodo et al. [105].

**Fig. 1:**
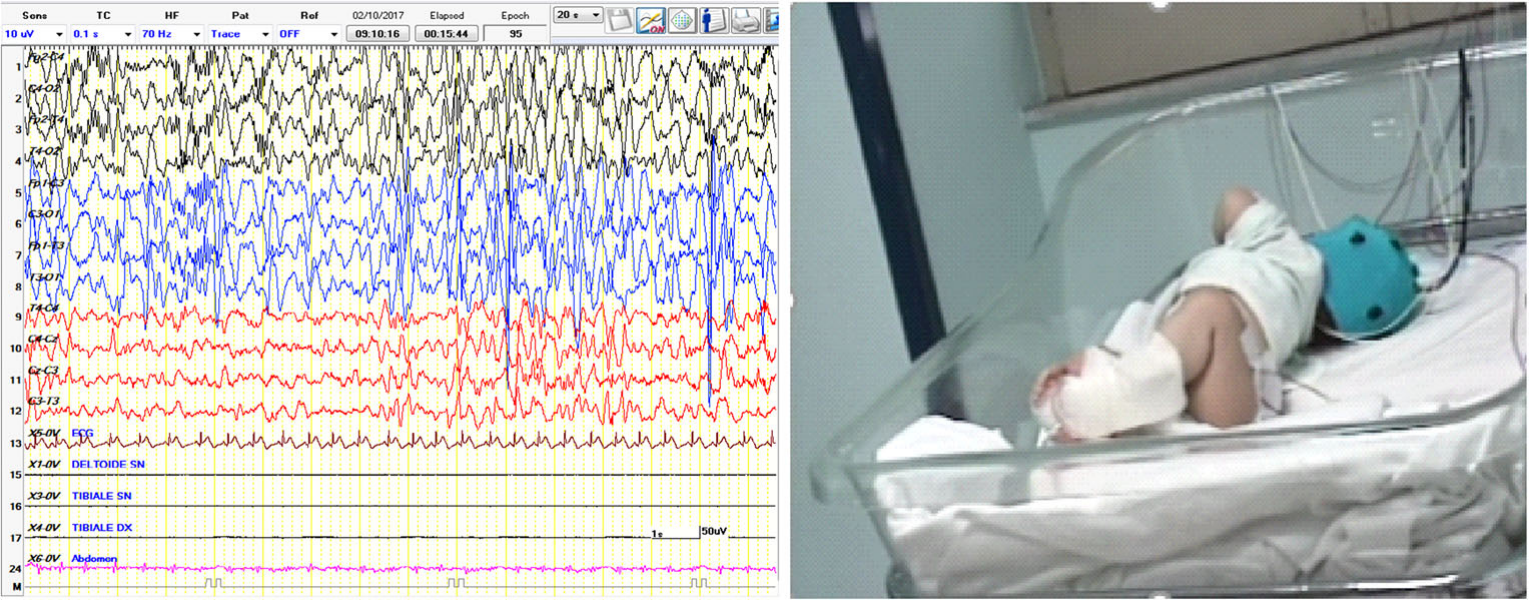
A six-month-old infant presenting slow movements of trunk rotation (“subtle spasms”)

**Fig. 2:**
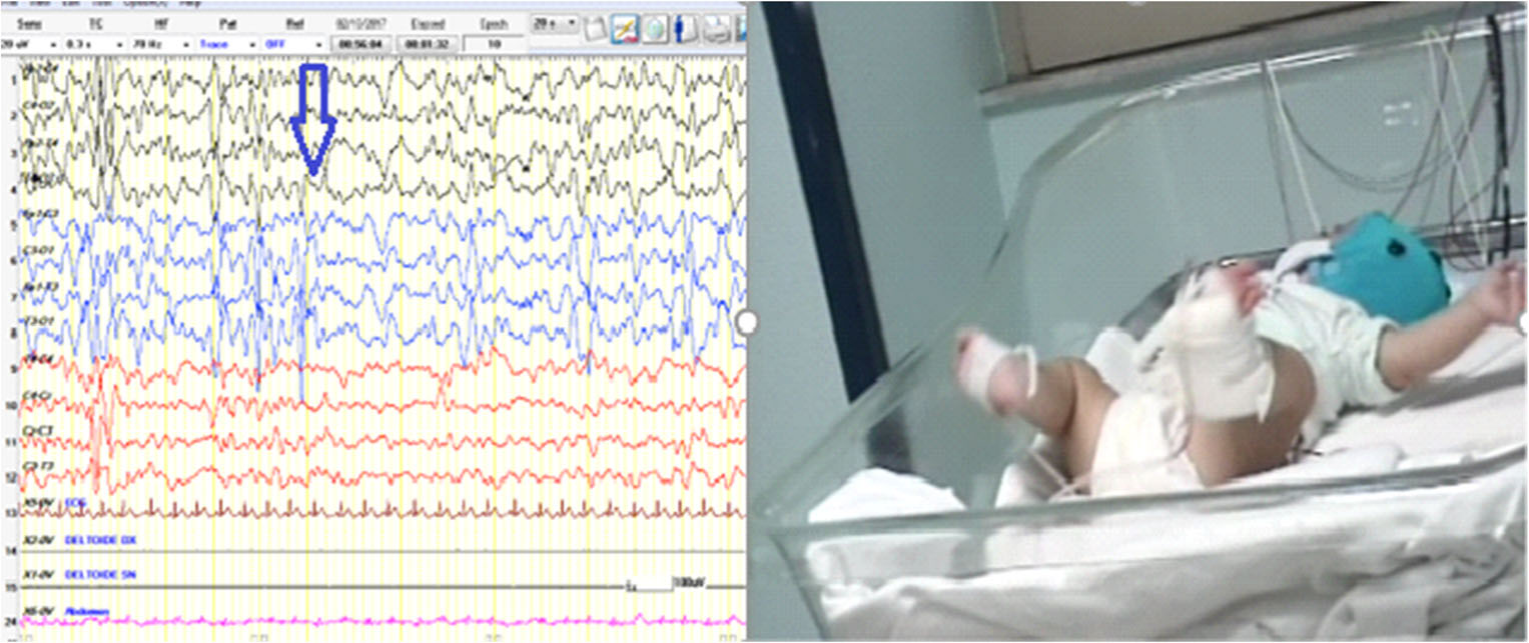
The same infant of Fig. 1 1 month later showing focal discharges and classical epileptic spasms

In general, the age of presentation of the spasms well correlates with the clinical and electroencephalographic patterns but there are some exceptions. Recently, some of us reported a child with Miller-Dieker syndrome and IS [106]: initially, the seizures were of the subtle spasms type with an EEG pattern of modified hypsarrhythmia (Fig. 1). A month later, the EEG pattern remained unchanged (focal discharges), whereas the epileptic spasms assumed the typical aspect of IS (Fig. 2). An analysis of infants with spasms in comparison to infants with other seizures types was conducted by Berg et al. [107]: according to this study, the age at onset of spasms was similar in infants initially presenting with typical spasms (6.1 months) vs. infants with spasms developing at (slightly) older ages (6.9 months); conversely, the age at onset of the other-than-IS seizure types was lower (4.7 months, *p* < 0.0001), demonstrating that the classical spasms develop around a given and well-defined age. Notably, no correlation between gestational age and age at onset of spasms was recorded [107].

## Atypical EEG patterns

Besides the classical hypsarrhythmic pattern with background chaotic activities and disorganization with multifocal sharp waves and spikes (interictal brain activity) and decrement background activity (ictal event), other EEG patterns may be observed during the course of the IS syndrome. These different patterns are defined as “atypical” or “modified hypsarrhythmia” and consist of asymmetric features, focal discharges, and semi-periodic burst-suppression [11]. Focal EEG activities have been recorded through scalp EEGs as ictal gamma and beta activity during IS by Nairai et al. [108].We personally recorded focal activity, in a child with Miller-Dieker syndrome, mainly localized in the occipital areas [106].

## Differential diagnosis and diagnostic approaches to children with presumed ISs

Two distinct clinical disorders may mimic the symptoms of ISs: (1) benign spasms of infancy usually manifesting in the first year of life; and (2) myoclonic epilepsy of infancy. Diagnostic assessment in children with ISs involves several aspects. Taking an accurate family history and performing a detailed general physical examination, particularly focused to get a full neurological assessment (including fundoscopy) but also aimed to exclude systemic anomalies involving the skin, face, heart, limbs, internal, and genital organs form the basis of a first diagnostic approach to a child with IS. A complete video-EEG recording is the next step and then, if the diagnosis of ISs is confirmed, further steps include ultrasound examination of the heart and internal organs and a magnetic resonance (MR) study of the brain (and the spine, when dictated by the clinical findings). In a retrospective review of 71 ISs cases, brain abnormalities at MR were found in 52/71 [109]: these 52 positive-MR children were divided into two subgroups, supposed (1) developmental and (2) acquired, both presenting with cortical gray and/or white matter abnormalities. Array-CGH analysis represent the first step for searching chromosomal abnormalities and/or deletions/duplications; further genetic testing should include oriented NGS analysis, or NGS analysis comprehensive for whole epilepsy gene panels; when available, WES and WGS should complete the genomic analyses, especially in cases negative to array-CGH and NGS. Metabolic assessment are mandatory when the disorder shows a rapid progression [17, 93–95].

## Treatment

**Fig. 3:**
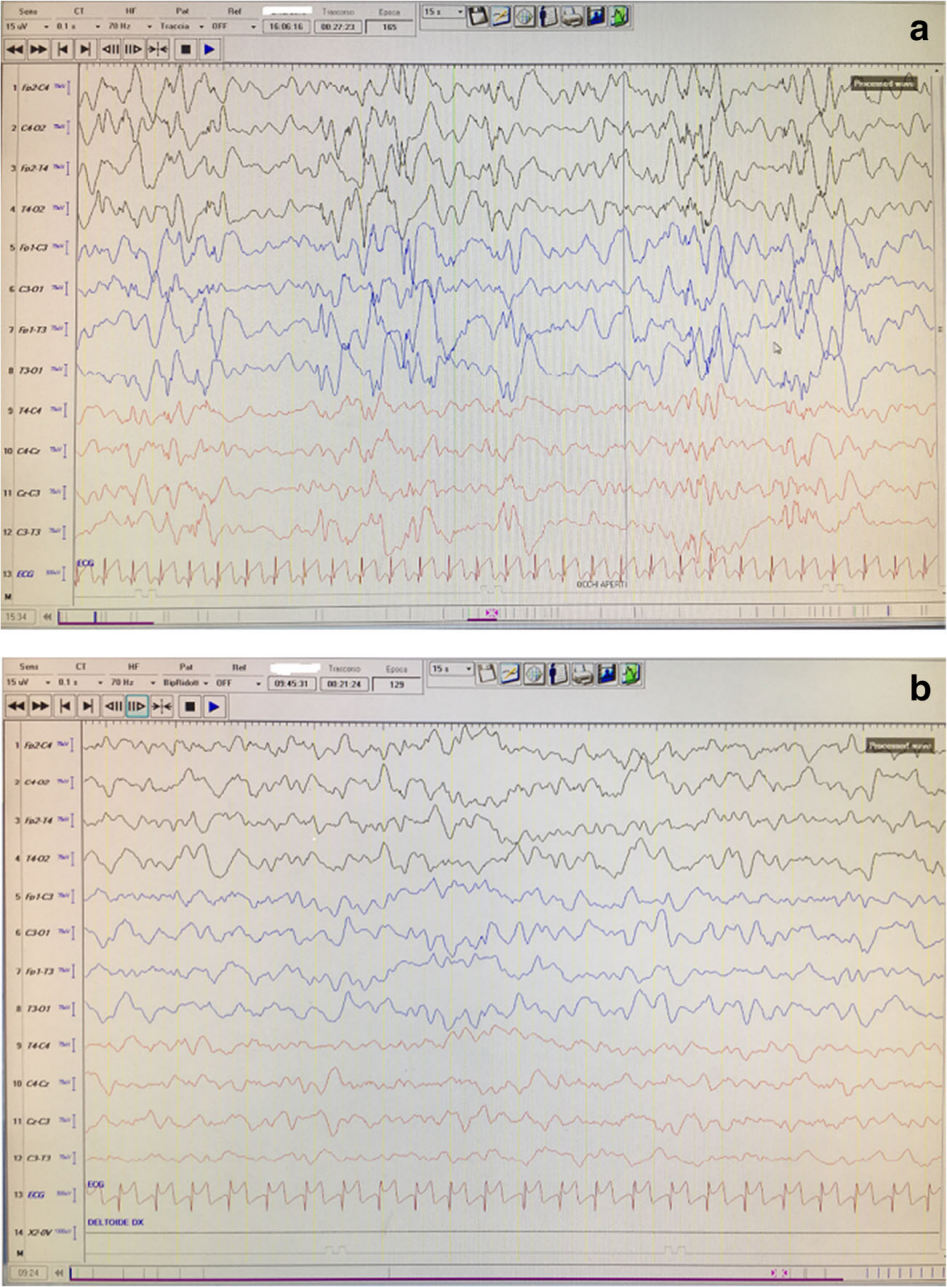
(**a**) Frame of video-EEG of a 6-month-old infant with pyridoxine-dependence before treatment with vitamin B6; (**b**) frame of video-EEG a day after treatment with pyridoxine

An early diagnosis and a shorter lag time to start treatment still represent the golden standards to get an effective response. Once the diagnosis of ISs is made, a therapeutic attempt should be started with a bolus of pyridoxine at the dose of 150 mg in 5–10 min, to exclude pyridoxine-dependent epilepsy: in this latter case, a rapid improvement of spasms and EEG abnormalities will be recorded within a few hours (Fig. 3).

The gold standard therapy for ISs consists in the administration of adrenocorticotrophic hormone (ACTH), vigabatrin (VGB), and corticosteroids [110, 111].

ACTH is used with a large variation of dosage, depending on the experience of each Institution. In a study on 200 ISs children, treatment with ACTH at the dose of 2–3 IU/kg/day was more effective as compared to treatment at the dose of 1.1–1.9 IU/kg/day (or 1 IU/kg/day or even 3.1–4 IU/kg/day) (*p* < 0.01) [112]. Most studies however, and the majority of personal experiences gained worldwide, suggest that low doses are as effective as high doses [113]. ACTH treatment should be stopped after 3–4 weeks after initiation of the therapy; however, as suggested by D’Alonzo et al. [114], treatment with prednisolone at the dose of 40–60 mg/day prolonged for 14 days has been considered effective and well tolerated. Potential side effects are immunosuppression and infections, hypertension, metabolic reactions, and renal failure [93, 110]. Prednisolone is supposed to act by regulating and improving the ISs-related immune dysregulation [110]. Wanigasinghe et al. [115] in a study conducted on a cohort of ISs children after 3 months of treatment recorded a better control of spasms in the ISs children initially treated with prednisolone as compared with children treated with i.m. ACTH. In other studies, however, no significant difference was found between children treated with ACTH vs prednisolone [116]. Pyridoxine, as additional therapy to ACTH or prednisolone, has been used with contradictory results. In a pilot study, carried out in 62 ISs children with comparable baseline clinical and EEG features divided in two groups: (1) oral prednisolone alone vs (2) combination of prednisolone with pyridoxine, no beneficial effects were recorded in group 2 [117].

Ketogenetic diet has been proposed as an alternative and effective treatment in ISs. The diet consists in an intake composed of high fats, adequate proteins, and low carbohydrates. Kossoff [118] reported effectiveness of this diet in ISs patients both in reducing/stopping the spasms and in normalizing the EEG. Good outcomes have been also achieved with a modified ketogenic diet using MCT oil—the ATKINS diet (MA), which consists of a combination of very low carbohydrate elements (medium chain triglyceride, at a dose of 10 g/day) and high fat food intake. This treatment is reported to produce a marked reduction of seizure in about 45% of affected children [119, 120].

Several classical and novel antiepileptic drugs have been proposed for treating ISs. The most common and effective drugs include nitrazepam, levetiracetam, sodium valproate, topiramate, zonisamide, rufinamide, clobazam, perampanel, and vigabatrim (VGB), used as monotherapy or in variable combinations [120]. Among these drugs, some are of more recent introduction in ISs treatment protocols and thus clear results has not yet been achieved. Monotherapy achieved fair good results in about 20–40% of patients [11]_._ VGB is widely used in the treatment of ISs. VGB is generally used at starting dosage of 50 mg/kg/day up to 150 mg/kg/day. In a study conducted in 221 ISs patients, treatment with low doses (18–36 mg/kg/day) was compared with treatment with high doses (100–148 mg/kg/day): spasms cessation were obtained in the group receiving high doses in 15.9% of cases vs 7% of patients with low doses (*P* = 0375): good EEG resolution was obtained in 30.8% in the group receiving high doses vs 13.2% of those with low doses [121]. Retinal toxicity is the most feared adverse effects of VGB, which is reported to occur in 21–34% of infants treated with this drug when treatment is prolonged for more than 6 months. More recently, this drug has been associated to reversible and asymptomatic signal changes in the thalami, basal ganglia, brainstem tegmentum and cerebellar nuclei at brain imaging [122]: these signal abnormalities have been named “VGB-associated brain abnormalities” (VABAM). Hussain et al. [123] by analyzing brain MR images of 257 ISs children found asymptomatic VABAM in 6/40 children treated with VGB: he hypothesized that the signal abnormalities were related to combination of high doses of VGB and the concomitant hormonal treatment. In patients taking low doses of VGB, no brain MR anomalies were recorded. However, a combination of ACTH and VGB has been demonstrated to be effective in reducing ISs and in improving the EEG anomalies [124, 125]. In a cohort of 66 children with ISs treated with VGB at progressive doses—from 50 mg/kg/day to 150 mg/kg/day—and high doses of prednisolone (60 mg/kg/day), 22/66 (33%) showed remission of spasms and a BASED score of < 2 after VGB alone; while 26 (39.4%) showed remission of spasms and a BASED score < 2 after the association of VGB and prednisolone [126]. In a report by the International Collaborative Infantile Spasms Study (ICISS), VGB in association with hormonal treatment vs. hormonal therapy alone did not result in improved developmental or epilepsy outcomes at 18 months [110]. Recently, a treatment with a VGB analogue 3-amino-4-difluoromethylenyl-1-cyclopentanoic acid (CCP-115) has been proposed in a 1-year-old ISs patient as an alternative treatment to VGB [127].

A single center, retrospective study was conducted in 24 patients with ISs: 10 patients were treated in monotherapy with sodium valproate (VPA); other 10 in association with clonazepam and 4 in association with nitrazepam: complete cessation rate and a 50% reduction of spasms were obtained in 45.8% of patients [128]. The duration to complete cessation of spasms was 70 days whilst relapses occurred in 18.2% [128]. Developmental delay has been recently reported in patients treated with VPA and has been related to the long use of the drug. Acute liver failure after VPA exposure has been associated to patients who present DNA-polymerase gene (POLG) mutations [129].

The importance of a second treatment after initial failure has been confirmed by a multicenter prospective study by Knupp et al. [130] who report a good result in one third of children with ISs after the use of a second medication. The efficacy of a second treatment in children with ISs is maintained to be linked to a different mechanism of action of the second drug. In our institution, we are used to start treatment with ACTH 3 UI/kg/day associated to VGB 50 mg/kg/day. The treatment with ACTH is stopped after 3 weeks while treatment with VGB is continued for about 6 months, and subsequently shifted with VPA or other antiepileptic drugs. Cannabinoid treatment for epileptic seizures has received careful interest [131]. At the moment no clear results about the efficacy and long-term safety in ISs patients have been reported [132].

Surgery treatment for ISs is rarely indicated and may be performed when well-documented focal epileptogenesis is recognized and when pharmacologic treatment has failed. Surgery treatment for ISs is one of attractive alternative in children with focal structural lesion. Surgical intervention is indicated when focal epileptogenesis is well documented through MRI, PET scan investigation in concordance to EEG abnormalities and when pharmacologic treatment has failed [108]. Treatment strategy for ISs have not changed so much during the last two decades. Standard first-line treatment with ACTH or prednisolone, and VGB, alone or in combination remains the most followed regime of treatment [17, 133–135].

## Specific emergent antiepileptic therapies in IS

Genetic researches are running for targeted drug therapies proposed for correcting the underlying molecular/cellular dysfunction. At the moment, there is a number of trials including rapamycin/everolimus, targeting the mTOR pathway, for the treatment of *TSC1/TSC2* [136]; memantine, targeting the actin protein *GRIN2 A* (NMDA) receptor [137]; and Retigabine (ezogabine) for the treatment of voltage-gated potassium channel (*KCNQ2*)-related ISs, caused by ring chromosome 20 abnormalities [138].

## Clinical outcome

In most patients, developmental delay, ranging from mild to severe degrees occurs prior to the onset of IS or, sometimes, may be concurrent or may follow the first ictal events. It must be noted, however, that it is not easy to recognize the delay at such early ages. Hypotonia, abnormal archaic reflexes, lethargy, poor reactivity may be clinical signs preceding the onset of IS: all these developmental features are strictly related to the serial epileptic seizures and to background molecular/cellular pathogenic events causing the ISs phenotype. Results of intellectual outcome at adult age in a cohort of 147 subjects affected by ISs according to conventional psychological test or based on educational status gave the following results: 25 children attended school normally with intelligence quotient (IQ) more than 85; 11 were slightly impaired with IQ 68 to 85; 36 with mild learning disability and IQ 40 to 60; 75 with severe impairment with IQ less than 40 [139, 140]. Association of ISs and autism spectrum disorder (ASD) has been frequently reported. Features compatible with ASD were historically observed in the West’ son at his older age [11, 15, 141]. In a meta-analysis study of Strasser et al. [142] ASD was reported in 19.9% in subjects with ISs compared to 4.7% of other types of epilepsy. ASD was diagnosed in 33 (13%) out 214 subjects with ISs [143]. In the group of 147 subjects a long time follow up 20–35 years after the IS a remission of the seizures was reported in a third of cases, a third had seizures with a daily or monthly frequency, and in the remaining third the seizures occurred with less frequency [140–144]. In a study of Krijgh et al. [145], at 8 weeks and 1 year of follow-up of 162 subjects 64 (40%) were seizure free. IS tend to disappear within 3 to 4 years of age and transition from IS to Lennox-Gastaut syndrome is reported to occur in 18% of cases [139, 140]. Fatal evolution was reported in 13% of the cases by Granstrom [146] and by Riikonen in a series of 214 children with ISs [147].

## Prognosis

Several factors influence the outcome of children with ISs. Poor response to treatment, evolution towards other epileptic syndromes, the degree of developmental delay and intellectual disability, behavioral disturbances including autism spectrum disorders, general clinical impairment, worsening secondary to side effects of treatment. The recurrence of seizures is regarded as a negative factor for poor prognosis leading to intellective disability [6, 7]. All the above factors could certainly contribute to a poorer outcome, however, according to our personal experience and opinion and to literature review, major negative effects in the prognosis of ISs are related to the underlying (molecular/cellular) etiological event causing the syndrome [148]. According to GulMert et al. [149], the most relevant prognostic factors are the etiology, the age at the time of presentation, and late and inappropriate treatments. Outcome is usually poor in cases of ISs related to severe brain malformations, post infectious diseases, and primary genetic causes [140]. Until now, the efficacy of treatment in ISs is limited, in most cases, when the pathogenic/etiologic event is severe. Targeted genetic treatments represent a future hope for a severe condition such as ISs.

## Conclusions and future directions

WS has evolved in each of the several aspects manifested by the patients affected by this severe disorder. Since the first report by West [1] several studies have been carried out aimed to solve the numerous enigma which this disorder hides. Many questions remain unclear and unsolved and suggest steps forward. Great advances have been obtained in the field of genetics which has allowed to identify new etiologic factors. Combined therapy has shown to give quit good results. In a next future the development of new therapies based on new or old molecules and the advances in molecular targeted therapy may represent the best way to avoid the harmful effects of this severe disorder.
